# Adiponectin through its biphasic serum level is a useful biomarker during transition from diastolic dysfunction to systolic dysfunction - an experimental study

**DOI:** 10.1186/1476-511X-11-106

**Published:** 2012-08-30

**Authors:** Mingqiang Fu, Jingmin Zhou, Juying Qian, Xuejuan Jin, Hongmin Zhu, Chunlin Zhong, Michael Fu, Yunzeng Zou, Junbo Ge

**Affiliations:** 1 Shanghai Institute of Cardiovascular Diseases, Department of Cardiology, Zhongshan Hospital, Fudan University, Shanghai, China; 2 Department of Medicine, Sahlgrenska University Hospital/Sahlgrenska, Gothenburg, Sweden; 3 Institutes of Biomedical Sciences, Fudan University, Shanghai, China

**Keywords:** Hypertension, Diastolic function, Systolic function, Adiponectin, Biomarker

## Abstract

**Background:**

Adiponectin is reported to relate with cardiovascular diseases, we sought to examine whether adiponectin is associated with disease progression of heart failure from hypertension in rats in comparison with other known biomarkers and echocardiographic parameters. Spontaneously hypertensive rats (SHR, *n* = 35), aged 1 month, were used and followed up to 18 months. High frequency echocardiography was performed both at baseline and every 3 months thereafter. Moreover, serum levels of N-terminal pro-natriuretic peptide (NT-proBNP) and interleukin-6 (IL-6) as well as serum level and tissue expression of adiponectin were determined at the same time as echocardiography.

**Results:**

The results clearly demonstrated time-dependent progression of hypertension and heart dysfunction as evidenced by gradually increased left ventricular mass index, NT-proBNP, IL-6 as well as gradually decreased cardiac function as assessed by echocardiography. Meanwhile, tissue and serum adiponectin decreased from 3 months and reached plateau until 12 months in parallel with decreasing of cardiac diastolic function. Thereafter, adiponectin levels increased prior to occurrence of systolic dysfunction. Adiponectin concentration is inversely related with NT-proBNP, IL-6 and E/E′ (correlation coefficient (r) = −0.756 for NT-proBNP, p < 0.001, -0.635 for IL-6, p = 0.002, and −0.626 for E/E′, p = 0.002, respectively) while positively correlated with E/A and E′/A′ (r = 0.683 for E/A, p = 0.001, 0.671 for E′/A′, p = 0.001, respectively). No difference for adiponectin distribution among visceral adipose tissues was found.

**Conclusion:**

Adiponectin through its biphasic serum level is a useful biomarker during transition from diastolic dysfunction to systolic dysfunction.

## Introduction

Adipose tissue has been long considered to be an energy-storage compartment before discovery of the various adipocytokines, such as adiponectin, leptin, plasminogen activator inhibitor type 1 (PAI-1), interleukin(IL)-1β, IL-8, IL-10, and so on [1,2]. All these adipocytokines exert multiple biological activities and significantly contribute to the regulation of the body’s homeostasis [3,4]. Notable among these is adiponectin, an adipose-tissue-specific plasma protein which is accounting for as much as 0.01% of total plasma proteins [5].

Adiponectin, also referred to as Acrp30/AdipoQ/apM1/GBP28, belongs to the collectin family [6] and contains four structural domains based on its primary sequence: an N-terminal signal peptide, a short hyper-variable region, a collagen domain, and a C-terminal globular domain homologous to C1q [7]. Basic researches and clinical studies have demonstrated that it not only exhibited anti-diabetic, anti-atherogenic and anti-inflammatory properties [8-10] but also had important protective effects at cardiovascular level [11]. However, the role of adiponectin in the development of cardiac diseases remains less clear than it does for metabolic disorders. Initial evidence from clinical studies showed that exposure to angiotensin II receptor blockers increased circulating levels of adiponectin. Moreover, the induction of hypertension with angiotensin II injection leads to a decrease in plasma adiponectin concentration concomitant with blood pressure elevation [12]. The association between adiponectin and hypertension is also evident in clinical studies by showing that hypoadiponectinemia is a risk factor for hypertension independent of insulin resistance and diabetes [13,14]. However, till to now, few studies address the time-frame changes of adiponectin during transition from hypertension to onset of cardiac dysfunction and heart failure. Therefore, our hypothesis is that adiponectin may serve as a useful biomarker for transition from left ventricular hypertrophy to cardiac dysfunction and heart failure. In this study, we examined whether serum and tissue adiponectin levels are associated with onset of heart failure from hypertension through left ventricular hypertrophy in rats in comparison with other known biomarkers for heart function and inflammation.

## Materials and methods

### Animals

Thirty five male spontaneously hypertensive rats (SHR) were purchased from Shanghai Laboratory Animal Center, Chinese Academy Sciences at 2 weeks of age. All rats were maintained in a specific pathogen-free room under conditions of temperature- and humidity- controlled with a 12:12-h light–dark cycle and given standard laboratory chow and tap water ad libitum. The starting age was 4 weeks. All of the animal experiments were performed in compliance with the Guide for the Care and Use of Laboratory Animals published by the US National Institutes of Health (NIH Publication No. 85–23, revised 1996) and was approved by the guidelines for animal research of Zhongshan Hospital, Fudan University.

### Experimental protocol

SHR were weighed, and systolic blood pressure (SBP) and heart rate (HR) were measured by a tail-cuff method (BP-98A, Softron, Tokyo, Japan) while conscious at 1, 3, 6, 9, 12, 15 and 18 months of age, respectively. After body weight (BW) and SBP data obtained, transthoracic high frequency echocardiographic studies were conducted. Thereafter the rats were sacrificed at each of the above time points to measure the levels of serum NT-proBNP, IL-6 and adiponectin, as well as the distribution and changes of adiponectin in different visceral adipose tissues.

### Echocardiographic studies

SHR were anesthetized with intraperitoneal injection of 50 mg/kg ketamine hydrochloride, and an additional 0.1-0.3 ml was given if needed. Rats were allowed to breathe spontaneously during the echocardiographic studies with a mean heart rate at 393 ± 18 bpm.

After being anesthetized, the rats were transferred to a heated platform and precordial region of the rats was shaved and warmed acoustic coupling gel was applied. Subsequently, transthoracic echocardiographic measurements were performed in the supine position with a commercially available echocardiographic system (Vevo770 High Resolution Imaging System, Visual Sonics, Toronto, Canada) using a 17.5 MHz transducer. The transthoracic echocardiographic probe was placed to obtain parasternal long and short-axis cardiac views. From the cardiac long axis, an M-mode trace of the LV was obtained, and heart rate (HR), LV end-diastolic diameter (LVEDD), LV end-systolic diameter (LVESD), LV posterior wall thickness (LVPW) and interventricular septum thickness in diastole (IVS) were measured following the American Society of Echocardiography guidelines [15]. The LV ejection fraction (EF) and fractional shortening (FS) were measured directly from the long-axis image. Mitral inflow was recorded at the tip of the mitral valve from a parasternal long-axis view using Pulsed-Wave Doppler Imaging. We measured maximal velocities of the E and A waves, isovolumic relaxation time (IVRT, defined as the interval between the aortic closure click and the start of mitral flow) and deceleration time of the E wave (DT, the interval between the peak early diastolic velocity and the point where the steepest deceleration slope was extrapolated to the baseline) [16]. E/A ratio was calculated. Using Tissue Doppler Imaging, both wall movement at the level of the lateral mitral annulus from the parasternal long-axis view and maximal velocity of early (E′) and late (A^′^) diastolic waves were recorded. E^′^/A^′^ and E/E^′^ were calculated. All measured and calculated indices are presented as the average of three consecutive cardiac cycles. The studies were performed by a trained echocardiographer who was blinded to the protocol. The gained images and data were stored and then downloaded to a magneto-optical disk for offline analysis.

### Serological studies

After echocardiography completed, adequate anaesthesia was achieved by additional intraperitoneal administration of ketamine hydrochloride. An incision was made in the abdomen, blood was sampled from the inferior vena cava and kept at −80°C for subsequent assay of NT-proBNP, IL-6 and adiponectin. Commercially available enzyme linked immunosorbent assay (ELISA) kits (ADL Biotechnology, Inc., USA) were used to measure serum NT-proBNP, IL-6 and adiponectin concentrations respectively according to the manufacturer’s instructions. Because the distributions of NT-proBNP and adiponectin were skewed, logarithmically transformed values were used for statistical analysis.

### Quantification of adiponectin and IL-6 mRNA expressions

After blood samples obtained, the abdominal incision was prolonged upward to the chest, and pericardial, perinephric and mesenteric adipose tissue samples were harvested from each SHR and quickly stored at −80°C for subsequent tissue study. Meanwhile, the LV was incised, retrieved and washed in 0.9% NaCl and then drawn up liquid by filter paper to get weighed for the calculation of left ventricular mass index (LVMI).

Total RNA was prepared by RNA-Trizol extraction (Invitrogen) and treated with DNase I (Takara). cDNA was synthesized using Reverse Transcription System (Promega, Madison, WI, USA) according to the manufacturer's instructions. Quantitative Real-Time PCR (qRT-PCR) analysis was performed on an iCycler system (Bio-Rad, Hercules, CA, USA) using SYBR GREEN I as a double-stranded DNA-specific dye according to the manufacturer's instructions (Applied Biosystems, Foster, CA, USA). Primers were designed as follows: 5^′^- CAG GTT GGA TGG CAG GCA TC-3^′^ (sense) and 5^′^-GTA AGC GGC TTC TCC GGG CT-3^′^ (antisense) for rat adiponectin, 5^′^-ACT TCA CAA GTC GGA GGC TT-3^′^ (sense) and 5^′^-AGT GCA TCA TCG CTG TTC AT-3^′^ (antisense) for rodent IL-6, 5^′^- CAG TGC CAG CCT CGT CTC AT-3^′^ (sense) and 5^′^-AGG GGC CAT CCA CAG TCT TC-3^′^ (antisense) for rodent GAPDH. All primers were obtained from Sangon Biotech (Shanghai) Co., Ltd. The expression levels of examined transcripts were compared to that of GAPDH and normalized to the mean value of controls.

### Western blot analysis

The adipose tissue samples were homogenized in lysis buffer (Cell Signalling Technology, Inc., USA) with protease inhibitor cocktail (Sigma Chemical Co., St. Louis, MO, USA). Protein content was determined by the BCA method. The same amounts of protein (50 μg) were separated with denaturing SDS 10% polyacrylamide gels. The membranes were immunoblotted with the primary antibodies at a 1:1000 dilution followed by incubation with secondary antibody conjugated with horseradish peroxidase (HRP) at a 1:2000–1:5000 dilution. Bands were visualized using ECL Western Blotting Detection kit (Amersham Pharmacia Biotech, Piscataway, NJ).

### Statistical analysis

All values are expressed as means ± standard deviation (SD). Group differences were analyzed by analysis of covariance (ANOVA). To compare multiple groups, Mann–Whitney U-test with Bonferroni correction was used. Bivariate analysis was performed to determine the correlation between plasma adiponectin levels and the other continuous variables. Group differences or correlations with p < 0.05 were considered as statistically significant. All analyses were carried out with SPSS16.0 statistical package for Windows (SPSS Inc, Chicago, Illinois).

## Results

### Body weight, systolic blood pressure and calculated left ventricular mass index

**Figure 1:**
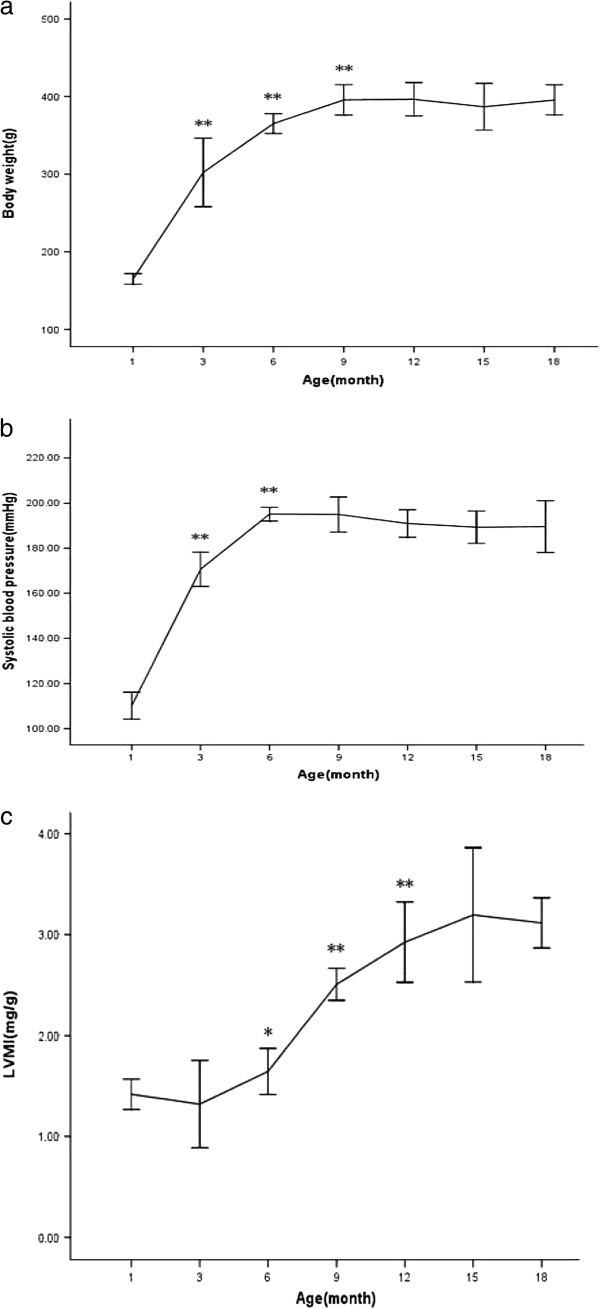
Changes in body weight (a), systolic blood pressure (b) and left ventricular mass index(c) for SHR. * p<0.05 vs. previous time point; ** p<0.01 vs. previous time point.

All rats survived. Body weight of SHR increased steadily with age and kept steady from 9 months to 18 months of age. There was significantly elevated SBP from 110 ± 5 mmHg at 1 month to 171 ± 3 mmHg at 3 months, and reached to 195 ± 3 mmHg at 6 months, henceforth remained plateau from 9 month to 18 month between 195 ± 7 mmHg and 190 ± 9 mmHg. LVMI became greater and greater along with the development of hypertension and aging (Figure 1).

### Echocardiographic parameters

Echocardiographic data are shown in Table 1. At 3 months of age, LV geometry of SHR demonstrated concentric hypertrophy associated with increased LVEDD, IVS and LVPW without change of LVESD. LVESD exhibited a steady increase at 9 months of age. Combined with progressively increased LVMI, an eccentric LV hypertrophy was overt. These observations are consistent with thickening of the walls between 3 and 18 months of age and a pattern of dilatation of the LV between 9 and 18 months of age.

**Table 1:** Echocardiographic data of SHR

	**Chamber and wall dimensions**				**Systolic function**	
**Month(s)**	**LVEDD(mm)**	**LVESD(mm)**	**IVS(mm)**	**LVPW(mm)**	**EF(%)**	**FS(%)**
1	3.79±0.29	1.71±0.09	1.28±0.18	1.14±0.08	90.87±0.95	62.32±1.05
3	4.79±0.22**	1.89±0.09	1.60±0.09**	1.60±0.14**	91.45±0.54	63.46±1.69
6	5.22±0.42**	1.95±0.07	1.79±0.11**	2.39±0.23**	90.67±0.65	62.73±1.29
9	5.51±0.38**	2.60±0.24**	1.83±0.12**	2.51±0.20**	91.15±0.69	62.89±1.94
12	5.61±0.25**	2.63±0.14**	2.05±0.20**	2.87±0.17**	91.68±0.76	63.40±1.64
15	6.17±0.40**††	3.61±0.16**††	2.08±0.10**	3.05±0.11**†	91.11±0.77	63.17±1.51
18	5.97±0.38**†	3.72±0.20**††	1.93±0.10**†	3.55±0.24**††	88.03±1.46**††	57.33±2.65**††
	**Diastolic function**					
**Month(s)**	**E/A**	**E′ /A′**	**E/E′**	**DT(ms)**	**IVRT(ms)**	
1	2.85±0.20	1.76±0.09	19.23±3.05	22.67±2.59	30.66±11.95	
3	2.78±0.26	1.72±0.09	27.12±2.06**	21.39±1.27	37.50±2.20	
6	2.26±0.43*	0.56±0.05**	29.66±3.64**	26.67±4.77*	39.14±6.54*	
9	2.15±0.34**	0.63±0.07**	31.65±3.00**	24.41±2.55*	37.22±13.07	
12	2.19±0.32**	0.55±0.09**	35.36±4.29**	24.17±4.02*	38.75±4.54*	
15	2.06±0.09**†	0.57±0.04**	36.93±1.31**	22.09±6.48	46.67±2.35**	
18	1.98±0.23**††	0.59±0.03**	39.82±4.41**††	26.33±3.31*	40.17±7.60**	

All data are means ± SD. * p<0.05 vs. 1 month; ** p<0.01 vs. 1 month; † p<0.05 vs. 12 month; †† p<0.01 vs. 12 month.

To examine LV systolic function of SHR, echocardiographic measurements of left ventricular ejection fraction (EF) and fractional shorting (FS) were obtained. Compared with 1 month, both EF and FS declined at 18 months of age, or by 3.38% and 8.75%, respectively, however, the decreases were both statistically significant.

Diastolic function was assessed by using Pulsed-Wave Doppler Imaging of mitral valve and Tissue Doppler Imaging of lateral mitral annulus. E/A ratio declined at 6 months of age, suggesting a trend toward a greater fraction of ventricular filling in late diastole. Of note, E^′^/A^′^ ratio decreased sharply at 6 months of age, and remained persistently below 1 during the rest period of study. E/E′ increased as early as at 3 months, which was in parallel with blood pressure elevation. There were trends of prolongation pertaining to IVRT and DT. Hence diastolic dysfunction occurs with establishment of hypertension and becomes apparent after 6 months of age.

### Serum adiponectin level inversely correlated with NT-proBNP and IL-6

**Figure 2:**
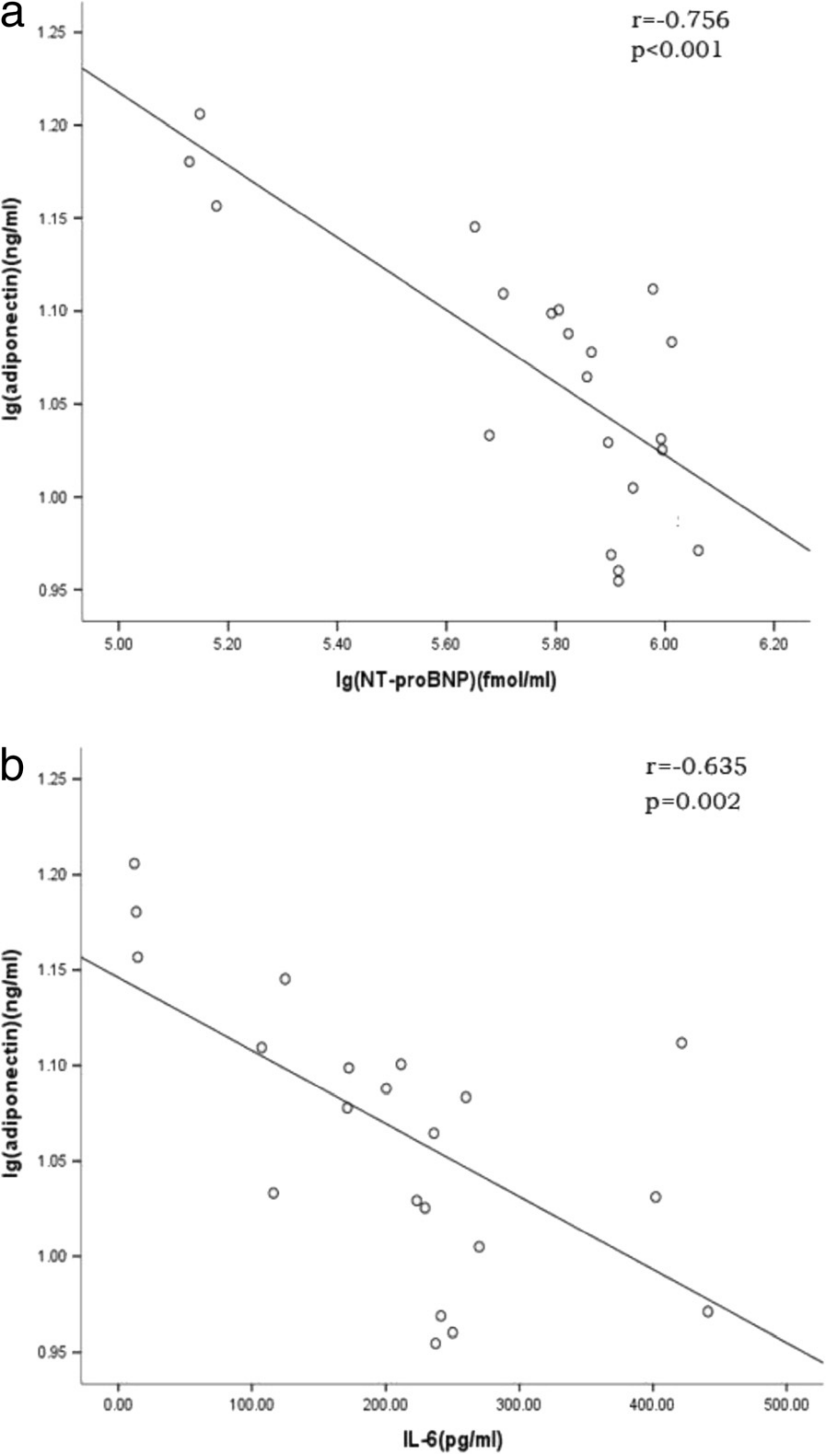
Relationship between serum adiponectin with NT-proBNP (a) and IL-6 levels (b).

Serum NT-proBNP level was elevated from 172.79 ± 4.32 fmol/ml at 1 month to 292.43 ± 7.59 fmol/ml at 3 months and then fluctuated between 339.43 ± 12.59 fmol/ml (6 months) and 407.97 ± 18.23 fmol/ml (18 months). IL-6 increased from 13.24 ± 1.25 pg/ml (1 month) to 116.09 ± 8.78 pg/ml (3 month) and eventually to 421.45 ± 19.49 pg/ml (18 month). However, adiponectin was initially decreased during first 3 months from 15.19 ± 0.86 ng/ml (1 month) to 12.54 ± 1.61 ng/ml (3 months) and then reached plateau until 15 months at 10.45 ± 1.52 ng/ml compared to that of 12 months at 9.64 ± 0.85 ng/ml. As shown in Table 2 and Figure 2, significant negative correlation was observed between serum adiponectin and NT-proBNP levels (r = −0.756, p < 0.001). And adiponectin level was also inversely correlated with IL-6 level (r = −0.635,p=0.002).

**Table 2:** Serum concentrations of NT-proBNP, IL-6 and adiponectin in SHR

**Month(s)**	**NT-proBNP (fmol/ml)**	**IL-6 (pg/ml)**	**Adiponectin (ng/ml)**
1	172.79±4.32	13.24±1.25	15.19±0.86
3	292.43±7.59**	116.09±8.78**	12.54±1.61*
6	339.43±12.59**	181.29±16.45**	12.25±0.29*
9	348.53±15.85 **	223.61±12.43**	11.64±0.95*
12	379.18±19.37 **	236.15±6.05 **	9.64±0.85**
15	386.52±19.69 **	260.05±9.90** †	10.45±1.52*†
18	407.97±18.23 **††	421.45±19.49** ††	11.01±1.81*†

All data are means ± SD. * p<0.05 vs. 1 month; ** p<0.01 vs. 1 month; † p<0.05 vs. 12 month; †† p<0.01 vs. 12 month.

### Correlations of serum adiponectin level with echocardiographic diastolic indices

As shown in Table 3, correlation analysis demonstrated significant positive correlation between lg(adiponectin) and E/A as well as E^′^/A^′^, while lg(adiponectin) was negatively correlated with E/E^′^. There was no correlation between lg(adiponectin ) with DT and IVRT.

**Table 3:** Correlations of lg(adiponectin) with echocardiographic diastolic indices

	**E/A**	**E'/A'**	**E/E'**	**DT(ms)**	**IVRT(ms)**
r	0.683	0.671	-0.626	0.287	-0.269
P value	0.001	0.001	0.002	0.207	0.238

### Distribution & expression of adiponectin and IL-6

**Figure 3:**
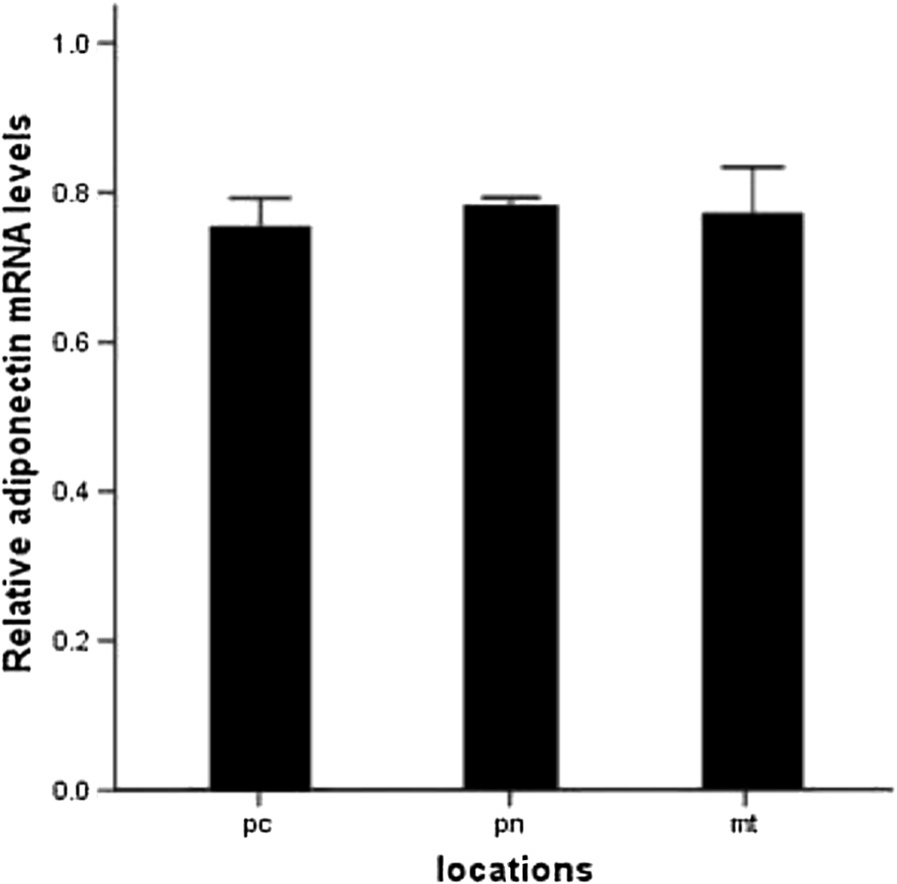
**Quantitative RT-PCR analysis of adiponectin mRNA among three different locations.** pc refers to pericardial adipose tissue; pn to perinephric adipose tissue; mt to mesenteric adipose tissue.

**Figure 4:**
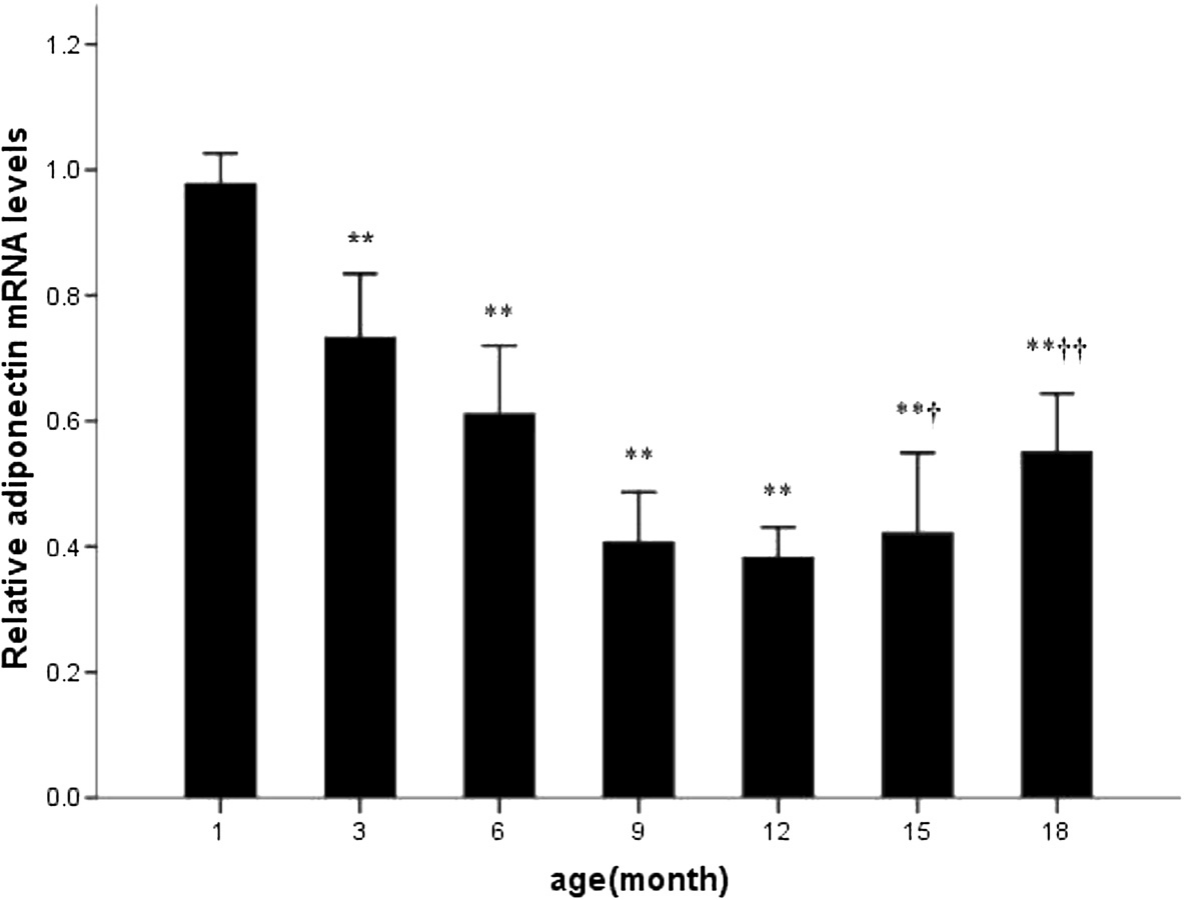
**Quantitative RT-PCR analysis of adiponectin mRNA with disease progression.** * p<0.05 vs. 1 month; ** p<0.01 vs. 1 month; † p<0.05 vs. 12 month; †† p<0.01 vs. 12 month.

**Figure 5:**
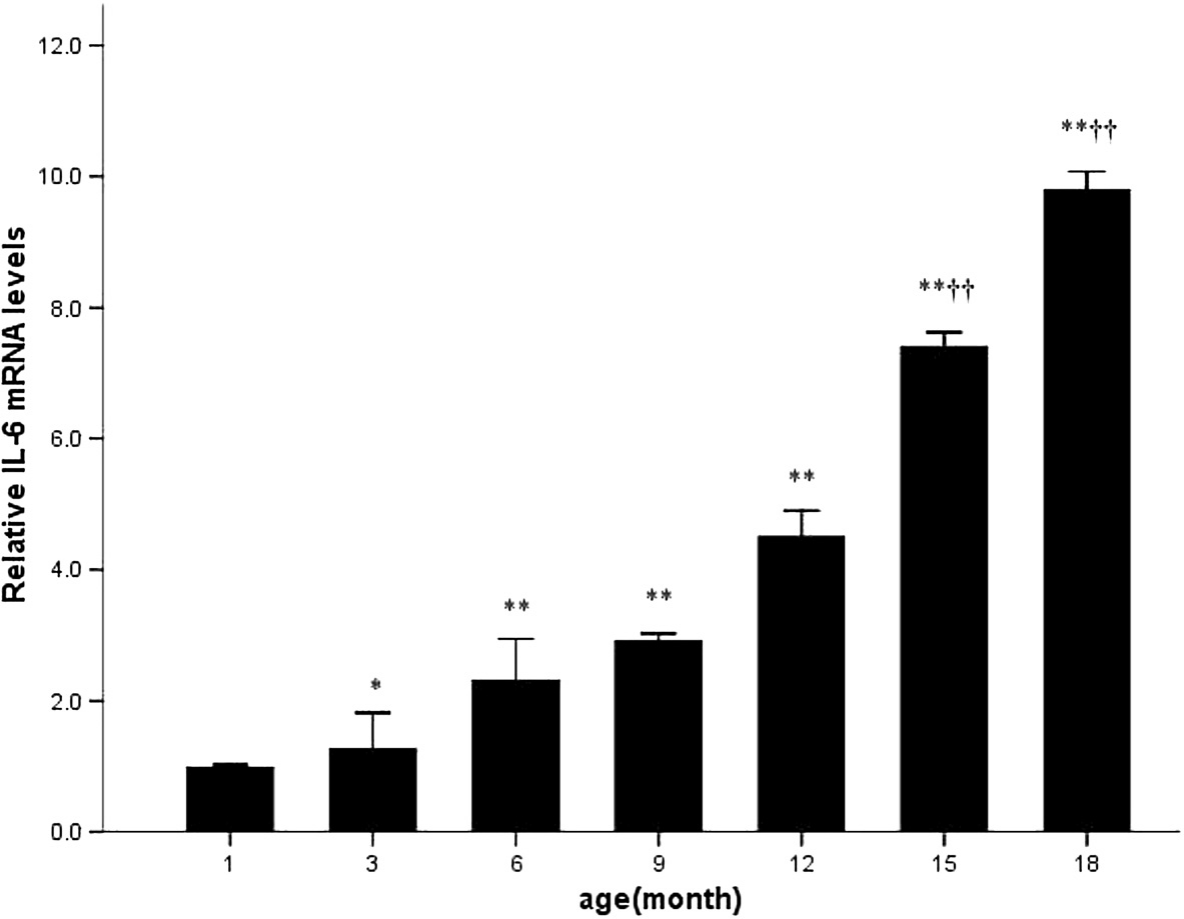
**Quantitative RT-PCR analysis of IL-6 mRNA with disease progression.** * p<0.05 vs. 1 month; ** p<0.01 vs. 1 month; † p<0.05 vs. 12 month; †† p<0.01 vs. 12 month.

**Figure 6:**
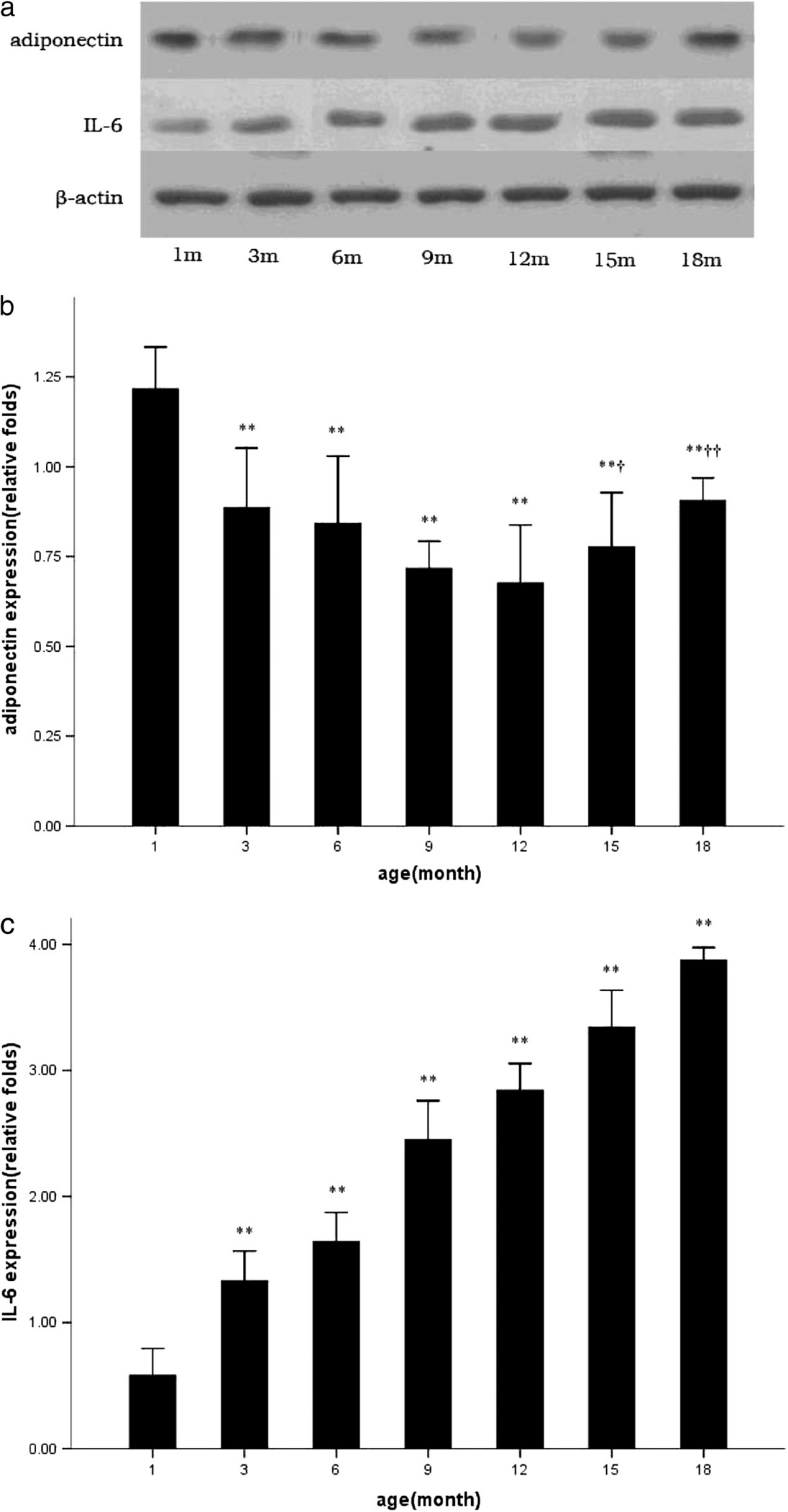
**Representative western blots (a) and qualification of adiponectin and IL-6 protein expressions with disease progression (b, c).** * p<0.05 vs. 1 month; ** p<0.01 vs. 1 month; † p<0.05 vs. 12 month; †† p<0.01 vs. 12 month.

There was no difference in distribution of adiponectin among pericardial, perinephric and mesenteric adipose tissues (Figure 3). However, with the development of left ventricular hypertrophy and cardiac dysfunction, adiponectin mRNA expression first decreased from 3 month to 12 month and then increased at 15 month thereafter (Figure 4). Meanwhile, IL-6 mRNA expression increased steadily with aging (Figure 5). Adiponectin and IL-6 protein transcriptions were in line with mRNA expressions, respectively (Figure 6).

### Intraobserver reproducibility

Reproducibility of the measurement of variables expressed as mean percent error was as follows: IVS, 1.44 ± 2.25%; EF, 5.83 ± 2.81%; FS, 6.19 ± 4.80%; E/A ratio, 0.66 ± 1.61%; E′/A′ ratio, 0.46 ± 1.26%.

## Discussion

The major findings of our study were that with advent of diastolic dysfunction since 3 month, adiponectin expression and serum concentration decreased simultaneously and increased at 15 month though systolic dysfunction appeared at 18 month. We also demonstrated no distribution differences of adiponectin among visceral adipose tissues.

SHR is a well established hypertension model similar to human hypertension such as the occurrence of LV hypertrophy followed by a transition to heart failure [17]. Therefore SHR has been regarded as a useful tool for studying the disease progression and mechanisms of LV hypertrophy and heart failure [18]. Echocardiography is one of the most widely used as a non-invasive technique to provide quantitative measurements of ventricular structure and function in humans and experimental animals. High-frequency transducers (17.5 MHz) was used in our study, which greatly overcame the difficulties of a small weight of around ~1 g with 2 mm LV wall thickness in rats and a rapid heart rate of 300–400 bpm, providing high resolution images for morphometric and functional measurements [19,20].

It is clear during the development of hypertension, alteration in LV geometry occurs as an early adaptation to increasing pressure and volume overload. In our study, during first 3 months, LVEDD, IVS and LVPW were increased whereas LVESD remain unchanged, indicating concentric hypertrophy. In the mean time cardiac function as assessed by echocardiography in terms of EF and FS was kept unchanged until 18 months. Meanwhile, diastolic dysfunction became overt from 6 month with decreased E/A ratio and E^′^/A^′^ ratio. However IVRT and DT values didn’t alter possibly due to high heart rate [21].

As a key functional abnormality in diastolic dysfunction and heart failure, abnormal diastolic filling pressure leads to a release of natriuretic peptides including NT-proBNP, which was released predominantly by the ventricles in response to stretch [22]. NT-proBNP levels increased significantly according to the severity of overall diastolic dysfunction, ranging from impaired relaxation to pseudonormal filling and restrictive filling, however, the role of NT-proBNP in patients with diastolic heart failure is still under investigation [23,24]. Although it has been found that NT-proBNP correlates with diastolic abnormalities in patients with reduced systolic function [25] and in patients with advanced forms of isolated diastolic heart failure [26,27], several Doppler echocardiographic studies found them not to be useful for the detection of mild diastolic dysfunction. The latter is associated with increased filling pressures at exertion only and will be missed by conventional Doppler echocardiography, which is performed at rest.

Documented data indicated that chronic inflammation is closely associated with the development of cardiovascular and cardiovascular-related disorders, such as hypertension, heart failure, etc. Adipokines secreted from adipose tissue, such as leptin and interleukin-6 (IL-6) showed significant contribution to the positive regulation of inflammatory.

It is reported that low levels of adiponectin was associated with a further progression of left ventricular hypertrophy in patients presenting with hypertension, left ventricular diastolic dysfunction and hypertrophy [28]. Our data are in line with such reports describing an association between decreasing levels of adiponectin serum concentration and increasing hypertension. In current study we determined multiple biomarkers including NT-proBNP, IL-6 and adiponectin at multiple time points during transition period from hypertension to heart dysfunction. Reason for choosing a combination of biomarkers such as NT-proBNP, IL-6 and adiponectin is to cover different pathophysiological mechanisms such as cardiac stress, inflammation and adipocytokines. Our results have shown that, being different from NT-proBNP and IL-6 which keep increasing during progression of heart dysfunction, adiponectin was decreased from 3 month when diastolic dysfunction occurred and then increased at 15 month prior to advent of systolic dysfunction which was apparent at 18 month. Therefore adiponectin is the biomarker indicating onset rather than progression of heart dysfunction. In contrast, NT-proBNP and IL-6 are biomarkers not only for onset of heart dysfunction but also progression of heart dysfunction. Though our animal model is mainly hypertension induced heart dysfunction rather than heart failure, as is evidenced in other studies [29,30], serum adiponectin level was elevated in chronic heart failure, our study also witnessed a trend.

Finally, adiponectin is produced exclusively by adipocytes [31] and its concentrations were predominantly determined by visceral fat [32]. However, up to now, difference in adiponectin gene expression in relation to various adipose tissue depots is still not fully elucidated [33]. Our study demonstrated no difference in adiponectin distribution, at least among pericardial, perinephric and mesenteric adipose tissues. However, adiponectin expression and serum level decreased with cardiac diastolic dysfunction and increased prior to systolic dysfunction while not in line with systolic dysfunction. We speculated the reasons might be as follows: (1) with disease progression, renal and hepatic functions might be impaired, and the clearance of adiponectin might also decrease [34,35], combined with relatively increased adiponectin expression, resulting in a prior serum elevation, which is partially consistent with our observations; (2) adiponectin is present in plasma in different isoforms: a large multimeric structure of high molecular weight and in a trimer and examer form, whereas the monomeric form is not found in peripheral circulation but only in the adipose tissue. Moreover, the lipolysis in adipose cells appears to be hormone-dependent, catecholamines and natriuretic peptides, besides insulin, exerting important effects [36]. (3) Circulating adiponectin concentrations can be regulated by various hormonal, nutritional and pharmacological factors. In heart dysfunction there are increased levels of IL-6 and TNF-α, as evidenced in our study, which might lead to decrease in serum adiponectin levels [37] and it is also reported adiponectin gene expression is reversibly downregulated by IL-6 [38]. The precise mechanism still needs further investigation.

In conclusion, we demonstrated that serum adiponectin level may be a useful biomarker during transition from hypertension to onset of cardiac dysfunction and adiponectin level is tempting to serve as a valuable indicator for early intervention. However, effectiveness and specificity are the two basic characteristics for a useful biomarker. The establishment of adiponectin as a valuable biomarker needs practical examination. Hence, whether and to what extent adiponectin could be used as a practical biomarker for cardiac function transition need more studies especially clinical data to support and prove and cautions should be take when making such conclusions.

## Competing interests

The authors declare that they have no competing interests.

## Authors' contributions

MF and JZ designed the study, carried out the experiments, and drafted the manuscript. JQ participated in the design of the study and helped to draft the manuscript. XJ performed the statistical analysis. HZ and CZ helped to carry out the experiments. MF participated in its design, and helped to draft the manuscript. YZ and JG revised the manuscript, conceived of the study, participated in its design. All authors read and approved the final manuscript.
